# Communicating to the Intellect, Heart, and Person: A Model Describing Participants’ Experience of Goals of Care Discussions Conducted During Acute Inpatient Care

**DOI:** 10.1089/pmr.2025.0020

**Published:** 2025-06-16

**Authors:** Joshua S. Lee, Katrielle Joy X.Y. Fu, Lynn Wiryasaputra, Celestine Z.Q. Lim, Paul Victor Patinadan, Joseph Y.J. Ong, Andy H.Y. Ho, Tricia S.H. Yung

**Affiliations:** ^1^ Department of Continuing and Community Care, Tan Tock Seng Hospital, Singapore, Singapore.; ^2^ Department of Geriatric Medicine, Tan Tock Seng Hospital, Singapore, Singapore.; ^3^ Institute of Geriatrics and Active Aging, Singapore, Singapore.; ^4^ Department of Geriatric Medicine, Woodlands Health Campus, Singapore, Singapore.; ^5^ Department of Palliative Medicine, Tan Tock Seng Hospital, Singapore, Singapore.; ^6^ Psychology Program, School of Social Sciences, Nanyang Technological University, Singapore, Singapore.; ^7^ Dover Park Hospice, Singapore, Singapore.; ^8^ Lee Kong Chian School of Medicine, Nanyang Technological University, Singapore, Singapore.

**Keywords:** patient care planning, palliative care, end-of-life care, grounded theory, patient-centered care

## Abstract

**Background:**

Goals of care (GOC) discussions align medical care with patients’ wishes. Many physician-associated barriers to GOC discussions have been identified, but there is little understanding of the lived experiences of patients and their nominated health care spokespersons (NHSs) who have participated in the discussion.

**Objectives:**

We aimed to describe the lived experience of participants of GOC discussions conducted during acute inpatient care and identify the features of well-conducted GOC discussions.

**Methods:**

We conducted a qualitative enquiry based on grounded theory, employing a social-constructivist approach and interpretivist research paradigm. Participants were purposively sampled from the medical oncology, geriatric, and palliative medicine departments of a tertiary hospital in Singapore. Data was gathered using semi-structured interviews.

**Results:**

A total of 24 interviews (16 NHS, 8 patients) were conducted. All participants were patients or NHS of patients who lived with a life-limiting illness (15 Cancer, 9 Non-cancer). The analysis yielded 2 major themes—*Preconceived health perceptions* and *Desired communications skills*—and 6 subthemes—*Elusive knowledge*, *Emotional undertones*, *Existential struggles*, *Explicit clarity*, *Embracing empathy,* and *Enhancing autonomy*. Well-conducted GOC discussions occurred when the participants’ health perceptions were satisfied by a complementary communication skill employed by the physician, based on the model “Communicating to the *Intellect*, *Heart,* and *Person*.”

**Conclusions:**

Well-conducted GOC discussions that facilitated consensus were discussions where the physician engaged the participant at three levels—the *Intellect, Heart,* and *Person*. Our model advocates for person-centered conversations where the views of the participant are heard and will provide insights to improve the conduct of GOC discussions.

## Introduction

The aim of a goals of care (GOC) discussion is to align recommended medical care with patients’ wishes that is based on their perception of health and treatment objectives. These discussions are conducted with the patient or a nominated health care spokesperson (NHS) who can make a best-interest decision for the patient. Well conducted GOC discussions result in a shared decision for the patient that improves medical care by ensuring better quality of life, reducing nonbeneficial treatment, and enhancing goal-consistent care.^1^ Despite the purported benefits, few patients with life limiting illness have had a GOC discussion.^2–4^

One reason for the lack of implementation of GOC discussions in routine acute inpatient care is the complexity of the discussion. GOC discussions have multiple components that cover illness understanding, goals and values, end-of-life planning, surrogate decision making, and advance directives.^5^ Though closely related, GOC discussions are different from advance care planning (ACP) and the associated frameworks proposed through the Serious Illness Care Program,^6^ and Respecting Choices model.^7^ These instruments focuses on the values, wishes or preferences for future care at end-of-life, to allow patients to plan when they lack decision making capacity.^8,9^ Alternatively, GOC discussions communicate overarching plans of medical care that are informed by patients’ underlying values and priorities, within the existing clinical context for current decision making^10^ and used to guide decisions about the use and limitations of medical interventions.^11^ In the setting of acute inpatient care, GOC discussions are often task specific, limited by time constraints and are susceptible to miscommunication error.

The physicians’ perspective of GOC discussions have been studied extensively and across multiple settings.^12,13^ Many physician-associated barriers have been identified—these include a perceived difficulty of a patient or NHS’s ability to understand treatment,^14^ lack of time^15^ and lack of prior relationship with the patient.^16^ Physician reported facilitators to good GOC discussion include interprofessional collaboration^17^ and a high health literacy of the participant.^15^ In contrast, there is little understanding of the lived experiences of patients and their NHS who participate in the discussion. Most studies surveyed participant responses to GOC discussion.^18,19^ Fewer studies have described their experience using a qualitative research methodology. Patients found the GOC discussion procedure focused and inappropriately discussed in their current health state.^20^ Most patients valued having their preferences heard, and communication strategies such as facilitating a safe and private setting were preferred.^21^

In Singapore, GOC discussions cover a range of topics including death and dying, prognostication, treatment plans, resuscitation plans, and caregiving preferences.^22^ To support its use during inpatient care, prompts are programmed into the electronic health system to remind health care providers to conduct these discussions. If a person lacks capacity for decision making, the GOC discussion is conducted with the NHS who is often an immediate relative, and the caregiver for the patient.^23^ A large majority of GOC discussions in the inpatient setting are conducted by junior physicians with varying experience in facilitating the discussion. This potentially accentuates some of the aforesaid physician-associated challenges that negatively impact the patient experience of the GOC discussion. Lastly, Singapore, being a multi-ethnic and multi-religious Asian country with traditional conservative cultural values may reflect a patient population with different outlooks to the GOC discussion from what has already been documented in literature.^24–27^ Thus, an in-depth analysis of the lived experience of the GOC discussion from the perspective of the participant, will provide insight to improve the conduct of GOC discussions.

The primary objective of this study is to describe the lived experience of patients’ and NHSs’ during the GOC discussion that was conducted in acute inpatient care. As a secondary aim, we explored participant’s perspective of a good and well-conducted GOC discussion and identified factors that they felt facilitated a consensus decision between them and the physicians.

## Material and Methods

### Study setting and design

The study was conducted from February 2022 to April 2024. Participants were purposively sampled from the medical oncology, geriatric, and palliative medicine department of a single tertiary hospital based on sociodemographic data and prognosis. Participants were patients with a life-limiting illness, or the NHS of a patient diagnosed with a life-limiting illness, who had undergone a GOC discussion during their inpatient stay and were above the age of 21 years. We excluded individuals who were unable participate in an interview because of physical impairments, who had moderate to severe cognitive impairment, and who were severely ill. Participants were sampled using a maximal variation sampling approach to ensure that the participants come from a variety of age groups, ethnicities, and religion, to explore the diversity of perspectives.

Data was gathered through one-one-one semi-structured interviews conducted by two independent interviewers with medical social work background using an interview guide (Supplementary Data) that required the participant to recount their experience of the GOC discussion and identify factors that led to consensus as well as their opinions on well-conducted discussions. Identified participants consented for an interview after the GOC discussion, and all interviews were conducted within a month of enrolment. The interview guide was modified based on emergent themes from concurrent data analysis using a constant comparison method.

### Data analysis

We employed a qualitative inquiry method led by grounded theory with a Straussian methodology^28^ to analyze the data, adopted a social-constructivist approach and interpretivist research paradigm. We analyzed the data collectively as a single participant group.

*NVivo (Release 1.0)* was used to manage the coding process. Line by line coding was first conducted by two researchers from the initial four transcripts. The research team met together to discuss these codes, and these were organized systematically into categories. This set of predetermined categories were used as a reference for subsequent transcripts. Joint coder meetings were held to discuss emerging themes and categories, and the interview guide was modified to explore new themes as they emerge. By constant comparative analysis, categories that were less meaningful were discarded from the final analysis, and existing categories were refined further. This process continued until theoretical saturation was reached. During axial coding, we identified how the categories interacted and linked them together. Finally, we constructed a theoretical model that explained the experience of participants for a GOC discussion. An audit trail of notes from team discussions was maintained to establish dependability of the study.

### Ethics

The National Health care Group Institutional Review Board approved the research protocol (DSRB reference number: 2021/00489). All participants provided informed consent for the study.

## Results

A total of 42 potential participants were invited to participate and 18 declined. We interviewed 24 individuals (16 NHS, 8 patients). All participants were suffering from or had loved ones suffering from a life-limiting condition (15 Cancer, 9 Non-cancer), most of whom (66.7%) had a prognosis of less than 1 year. The average age of the participants was 56.5 years old. Most participants were of Chinese ethnicity, completed tertiary education, earned less than SGD $5,000 per month and subscribed to an organized religion (Table 1).

**Table 1.** Cohort Characteristics and Demographics

	Cohort *N* = 24
Age, years	56.5 ± 16.5
Relationship to patient	
Nominated health care spokesperson	16 (66.7)
Patient	8 (33.3)
Gender	
Male	10 (41.7)
Female	14 (58.3)
Marital status	
Married	17 (70.8)
Single/widowed/divorced	7 (29.2)
Ethnicity	
Chinese	19 (79.2)
Malay	4 (16.7)
Indian	1 (4.2)
Faith	
Agnostic/Atheist	6 (25)
Buddhism/Taoism	6 (25)
Christian/Roman Catholic	7 (29.2)
Islam	4 (16.7)
Hinduism	1 (4.2)
Education level	
Primary	1 (4.2)
Secondary	5 (20.8)
Diploma	8 (33.3)
Degree	10 (41.7)
Employment status	
Employed/self employed	14 (58.3)
Unemployed	10 (41.7)
Monthly income^a^	
Less than SGD $5,000 a month	16 (66.7)
SGD $5,000- SGD $10,000 a month	5 (20.8)
≥SGD $10,000 a month	3 (12.5)
Diagnosis of patient	
Cancer	15 (62.5)
Non-Cancer^b^	9 (37.5)
Physician-estimated prognosis	
Less than 3 months	11 (45.8)
Between 3 and 6 months	2 (8.3)
Between 6 months and 1 year	3 (12.5)
More than 1 year	8 (33.3)

All values expressed as Mean ± Standard Deviation or N (%).

^a^ Monthly Income: In Singapore, median monthly income per household member for the year 2024 is SGD $3,615.

^b^ Non-Cancer: Advanced dementia with recurrent infection, heart failure, infective endocarditis that failed treatment, Stage V chronic kidney disease not for renal replacement therapy.

The analysis yielded 2 major themes [(1) Preconceived health perceptions and (2) Desired communications skills] and 6 subthemes [(1a) Elusive knowledge (1b) Emotional undertones (1c) Existential struggles, (2a) Explicit clarity (2b) Embracing empathy (2c) Enhancing autonomy] (Table 2).

**Table 2.** Major Themes About the Goals of Care Discussion

Theme 1: Preconceived health perceptions	
Subtheme 1a: Elusive knowledge	
Recognizing expertise	Anticipatory care
Because they are doctors and they are there to do their jobs, so we have to put the trust in them, we cannot be suspicious. We have to trust them because they are doctors-so if they tell me something, I have to listen to them. (GOC 10, NHS)	I think they were really prescient, and I think they were very pre-emptive… [they] tell you what could be up ahead and [they] want to get you ready. (GOC 5, NHS)
Subtheme 1b: Emotional undertones	
Persistent anxiety	Prevalent negativity
Whatever you say the next sentence, nothing gets in the ear. Your head is in a spin. Already for me, my head is in a spin. So, some words I caught, some words I missed right. (GOC 26, Patient)	I come from a point of view where I appreciate the practicality and the point of it… the reality of the situation you know… rather than rose-tinted picture to give us hope. (GOC 22, NHS)
Subtheme 1c: Existential struggles	
Perceived invincibility	Recognized futility
I said Ok. I mean, in life, you wouldn’t expect this thing to happen to you, you know, people say you live up to a certain age, you die… But you don’t think about someone having to look after you, someone having to care for you. (GOC 26, Patient)	There’s nothing to be done about it, you have to go. I have handled everything. I’m 70. I can go, my kids are all big, there’s nothing left to deal with. (GOC 12, Patient)
Theme 2: Desired communication skills	
Subtheme 2a: Explicit clarity	
Demystifying Jargon	Mitigating Complexity
Explain in layman term what are they, what are they going to do or what’s going to happen. At the same time, step-by-step, like, this is what’s going to happen. A, b, c, d. (GOC 14, NHS)	Yeah, help me break down my problems first, tell me what is the most immediate thing, right? Tell me, because of these complications, these are the potential crossroads you might be facing… help me break down the problem. (GOC 5, NHS)
Subtheme 2b: Embracing empathy	
Reflective listening	Shared struggles
He (the doctor) listens to you. If sometimes I’m trying to be positive, sometimes when you talk to him face to face, you will get emotional and he will tell you we don’t have to think too much, we just go step by step. (GOC 11, Patient)	I mean if you are already on the ship already, on this medication today and I will be seeing him next week. I will see him next week for blood test then decide what to do next. (GOC 1, Patient)
Subtheme 2c: Enhancing autonomy	
Respecting wishes	Informed choices
You can feel the respect he has for you. When he hears your opinion, he will say ok, I support your decision. Yes, he respects me quite a bit, he will always explain the treatment process, and let me have my own views. (GOC 19, Patient)	The doctors have laid out all the possibilities very clearly, they have told us what we can do, what we cannot do, yeah. I can’t, really ah, I think it’s been very well done, I can’t think of any better way to handle this (GOC 5, NHS)

GOC, goals of care; NHS, Nominated health care spokesperson.

### Theme 1: Preconceived health perceptions

We identified three perceptions of health that shaped the participants’ experience of the GOC discussion. These perceptions affected the way the participant interacted with the physician, informed their opinions regarding the content of the discussion and shaped the decisions that were made.

**Elusive knowledge.** We identified that participants lacked knowledge to make medical decisions and accrued this information during the GOC discussion. It manifested as two behaviors. First was reliance on the medical expertise of the physician to facilitate decision making. This was characterized by an attitude of deference to the opinion of the physician, as expressed in the quote below:

*Because they are doctors and they are there to do their jobs, so we have to put the trust in them, we cannot be suspicious. We have to trust them because they are doctors - so if they tell me something, I have to listen to them*. (GOC 10, NHS).

This posture of deference was a response to a deficiency of knowledge regarding medical treatment, coupled with the complexity of medical decision-making that was exacerbated by time constraints needed to make these decisions. Thus, to reach a quick resolution, participants defaulted to the professional judgement of the healthcare provider.

Second, a desire for knowledge was displayed as an expected tenor of anticipatory and prescient care. Participants viewed medical care as a predictable, controlled process. Such an opinion resulted in a desire for preparedness, which was demonstrated by this participant during the GOC conversation he experienced:

*I think they were really … prescient, and I think they were very pre-emptive… [they] tell you what could be up ahead and [they] want to get you ready.* (GOC 5, NHS)

We observed that the lack of knowledge caused apprehension, and participants sought personal understanding through acquisition of information during the GOC discussion.

**Emotional undertones.** Challenging conversations, such as those concerning GOC, are shaped by emotions. We found that emotions of anxiety and negativity predominated the discussions.

We discovered that anxiety had a paralyzing effect on the participants, leading to indecision. These emotions were impulsive and occurred when bad news was delivered or when there were urgent decisions to be made. One participant described the gyroscopic nature of the anxiety experienced during the GOC conversation as such:

*Whatever you say the next sentence, nothing gets in the ear. Your head is in a spin. Already for me, my head is in a spin. So, some words I caught, some words I missed right*. (GOC 26, Patient)

Accompanying the sense of anxiety was a prevalent posture of negativity that encompassed feelings of hopelessness and despair. Unlike anxiety which was experienced contemporaneously as the conversation developed, the sense of negativity preluded the discussion and was borne out of past experiences with illnesses. This resulted in a somber stoic disposition amongst many participants.

*I come from a point of view where I appreciate the practicality and the point of it… the reality of the situation you know… rather than rose-tinted picture to give us hope.* (GOC 22, NHS).

These tendencies toward negativity and anxiety slanted the experience of GOC discussions towards a more pessimistic timbre.

**Existential struggles.** Many participants endured prolonged illness and engaged in deep reflection on the meaning of their existence in relation to their health condition. Two contrasting opinions regarding end-of-life were identified.

One opinion was a sense of perceived invincibility regarding their current health situation. This was borne out of a state of denial; a conviction that their health condition was curable and a rejection of possible limitations of medical treatment. In the GOC discussion, this manifested as shock when hearing bad news, such as that expressed by this participant who could had trouble appreciating her mortality:

*I said Ok. I mean, in life, you wouldn’t expect this thing to happen to you, you know, people say you live up to a certain age, you die… But you don’t think about someone having to look after you, someone having to care for you*. (GOC 2, Patient)

The second opinion was a sense of recognized futility. Participants who adopted this posture were aware of the limitations of medical intervention and were willing to accept ceilings of care more readily when these were highlighted during the GOC discussion. Participants who held such an opinion would assume positions of acceptance as demonstrated by the participant below:

*There’s nothing to be done about it, you have to go. I have handled everything. I’m 70. I can go, my kids are all big, there’s nothing left to deal with.* (GOC 12, Patient)

We found that these contrasting opinions provided the foundational values on which medical decisions were made and were the basis from which the participants derived their choices on their GOC.

### Theme 2: Desired communication skills

All participants expressed preferences for certain communications skills to be demonstrated during the GOC discussion. These communication skills were preferred as they addressed the aforesaid participants’ health perceptions. We distilled 3 desired communications skills.

**Explicit clarity.** A challenging aspect of GOC discussions was the need to communicate complex medical information in a short time. Clear communication techniques were favored as it aided the participants to understand information better.

Clarity was exhibited when the physician demystified jargon. Many participants found it challenging to understand medical jargon and expected physicians to explain complex medical language in understandable terms:

*Explain in layman term what are they, what are they going to do or what’s going to happen. At the same time, step-by-step, like, this is what’s going to happen. A, b, c, d…* (GOC 14, NHS)

Participants also preferred that the physician mitigate the complexity of medical decision making. Techniques employed to simplify complexity included the use of visual aids during the GOC conversations, such as the use of graphics or diagrams. By doing so, the physician was able to summarize complex decisions into simpler, more manageable processes:

*Yeah, help me break down my problems first, tell me what is the most immediate thing, right? Tell me, because of these complications, these are the potential crossroads you might be facing… help me break down the problem.* (GOC 5, NHS)

Thus, communicating with explicit clarity allowed the physician to bring the participant through the pros and cons of medical treatment, thereby facilitating better conversations.

**Embracing empathy.** The ability to engage in empathetic conversation with a warm and embracing demeanor was identified as a crucial communication skill.

Reflective listening was a way to demonstrate empathy. This allowed the physicians to intuitively address the emotional needs of the participant, resulting in calm and reassurance as exhibited below:

*He (the doctor) listens to you. If sometimes I’m trying to be positive, sometimes when you talk to him face to face, you will get emotional and he will tell you we don’t have to think too much, we just go step by step.* (GOC 11, Patient)

Empathy was also demonstrated when the physician displayed a sense of shared struggles with the participant through the challenges of decision-making. An example was from a patient who likened his experience with his physician to a journey on a ship:

*I mean if you are already on the ship already, on this medication today and I will be seeing him next week. I will see him next week for blood test then decide what to do next.* (GOC 1, Patient)

We observed that displays of empathy provided resolution to many of the emotional undertones that were experienced by the participant during the conversation.

**Enhancing autonomy.** Finally, participants wanted discussions that enhanced their individual autonomy. It was common that participants in GOC discussions had different opinions from that of the physician. We identified that many participants wanted their physicians to respect their wishes, even when they differed.

*You can feel the respect he has for you. When he hears your opinion, he will say ok, I support your decision. Yes, he respects me quite a bit, he will always explain the treatment process, and let me have my own views.* (GOC 19, Patient)

In contrast, conversations techniques that did not acknowledge the opinions of the participants made them feel disengaged from the GOC discussion, resulting in greater conflict and dissatisfaction.

Similarly, autonomy was enhanced by ensuring comprehensive disclosure of medical options and treatment plans so that participants can make informed choices.

*The doctors have laid out all the possibilities very clearly, they have told us what we can do, what we cannot do, yeah. I can’t, really ah, I think it’s been very well done, I can’t think of any better way to handle this.* (GOC 5, NHS)

This allowed the participant to feel that there was no hidden agenda during the GOC discussion and led to a sense of self-empowerment when decisions had to be made.

### Theory — communicating to the “intellect, heart, and person”

**FIG. 1.**
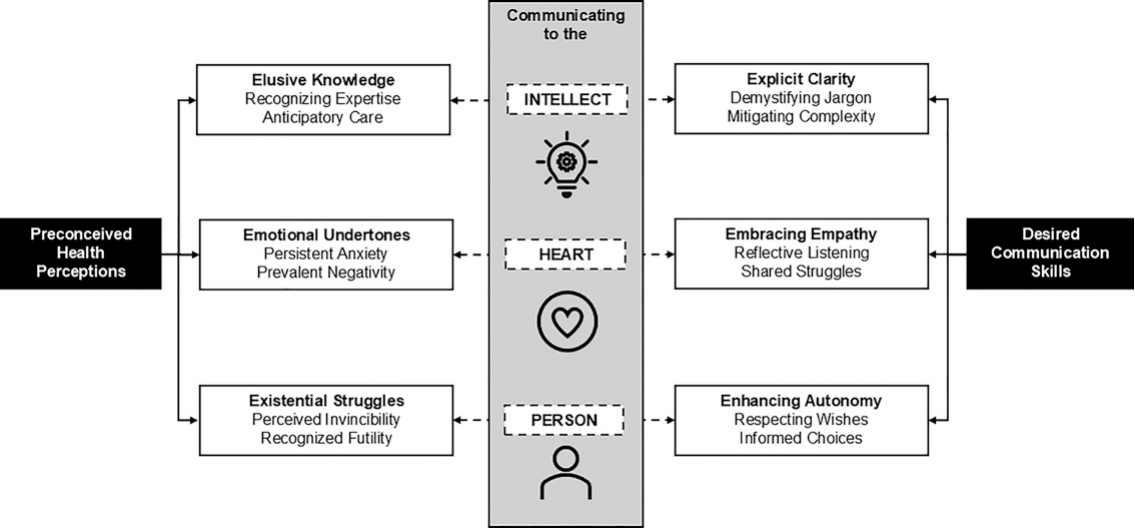
Communicating to the intellect, heart, and person: A model for well-conducted goals of care discussions.

Participants described GOC discussion as an interaction between the Preconceived Health Perception of the participant and the Desired Communication Skills demonstrated by the person conducting the discussion and surmised the theory-“Communicating to the *Intellect*, *Heart,* and *Person*” (Fig. 1). There were three domains that the interactions occurred. First, there was a component of the discussion that appealed to the ‘*Intellect*’. In this domain, the participants’ desire for knowledge was resolved by physicians who distilled complex medical information into clear, understandable terms for the recipient. Second, the emotive aspect of the conversation—the *‘Heart*’—needed to be addressed. In well-conducted GOC discussions, the strong emotional undertones were tempered by an embracing empathy that provides reassurance to the participant. Lastly, the values that shape the opinion of the participant need to be respected by enhancing the autonomy of the participant. This allowed the individuality of the ‘*Person*’ to be esteemed. GOC discussions were considered well-conducted when a participant’s perceptions were satisfied by a complementary communication skill employed by the physician conducting the discussion.

## Discussion

### Main findings

We studied the participants’ experience of GOC discussion conducted during acute inpatient stays and theorized that the GOC discussion was an interplay between the participants preconceived health perception and the desired communication skills they expected from their physicians.

### What this study adds

Other models for challenging communication exist, such as SPIKES,^29^ REMAP,^30^ and the GOOD framework.^31^ These tools adopt a dialectical method of argumentation,^32,33^ focusing on form and procedures to achieve consensus. They are constructed from the perspective of the physician, resulting in a list of rules and paradigms, that if followed, convinces the participant of the physician’s viewpoints. Such conversation aids put the physician and the participant on diametrically opposing sides, adopting a conflict resolution model to bring about consensus. These tools lean heavily on the rational component of the conversation and de-emphasize the subjective and emotional domains that formulate these discussions.

In contrast, our model is a 3-factor model that comprises a cognitive, emotional and intuitive domain. Such an approach to communication can be traced back to the Aristotelian persuasive traditions of *logos*, *ethos*, and *pathos* (rational argumentation, the speaker’s credibility and reliability, and the appeal to emotion).^34^ Rather than outline a process, our model of communicating to the *Intellect*, *Heart,* and *Person*, recognizes the subjective and intuitive components of the GOC discussion that is lacking in other models.

Person-centered communication is increasingly recommended for GOC discussion.^35^ Our model represents a person-centric model, because it is constructed from the participants’ perspective and values, and enables the patient in making health decisions that are concordant with these goals.^36^ It contains recognizable aspects Person-centered communication such as exchanging information, fostering healing relationships, recognizing and responding to emotions, managing uncertainty, making decisions, and enabling patient self-management.^37^ Our model proposes a collaborative person-centered approach, putting the needs of the participant, rather than agenda of the physician, at the core of the discussion.

Our study highlighted two interesting findings. First, the location where the GOC discussion was conducted was not a prominent theme in our study. This differs from conventional practice where the setting of the conversation plays an important role in the GOC discussion.^21^ We observed that in conversations where the physician could demonstrate empathy and clarity, the setting of the conversation was unimportant. We postulate that the setting was a vehicle in which empathy was demonstrated, and physicians who could empathize did not require an appropriate setting as a precondition to enhance their conversation. Second, the high regard that our participants have for the opinion of the medical professions is unique. We attributed this to differences in the cultural context in which this study was conducted which may lead to the difference in opinion that was identified.

### Limitations of the study

Our study had the following limitations. First, participants who had better experiences would be more willing to come forward to participate in the interview. Consequently, we may not have captured sufficient data from participants who had negative experiences. Second, the sample size for this study is insufficient to analyze differences in perspectives between subpopulations of participants who were sampled. Lastly, participants were selected from medical disciplines that conduct GOC discussion as part of routine care. Therefore, participants may have participated in GOC discussions that were conducted by physicians who are more practiced, and our findings might not be generalizable to discussions that are conducted by physicians with limited expertise in facilitating GOC discussion.

### Recommendations

We recommend the following for future studies and application. First, the model can be enhanced with discussion aids and employed in an implementation study to analyze if it would lead to increased participant satisfaction in GOC discussions. Second physician feedback when using the model can be harnessed to understand it’s practicability in real world GOC discussions. Lastly, the model can be used to structure training programs and inform the way physicians are taught to organize and structure GOC discussions, thereby resulting in a holistic ken when it comes to care.

## Conclusion

In conclusion, well-conducted GOC discussions that facilitate consensus are discussions where the physician engaged the participant at three levels—the *Intellect, Heart,* and *Person*. Our model advocates for person-centered conversations where the views of the participant are heard.

## Abbreviations Used

ACP: Advanced Care Planning
GOC: Goals of Care
NHS: Nominated Healthcare Spokesperson
