# Supplementary material for: Communicating to the Intellect, Heart, and Person: A Model Describing Participants’ Experience of Goals of Care Discussions Conducted During Acute Inpatient Care
Source: Palliat Med Rep. 2025 Jun 16;6(1):356–64. doi: 10.1089/pmr.2025.0020 (PMC12410331; doi:10.1089/pmr.2025.0020)
Supplement: Supplementary Data [file pmr.2025.0020_supplementary_data.docx]

**Online Supplement:**

Survey and In-depth Interview Guide

| **Part 1: Demographic Data**  ***To be completed by research team and /or interviewer if incomplete*** | |
| --- | --- |
| I am the | 1. Patient 2. Patient’s loved one; Specify relationship: __________________ |
| Age of Participants |  |
| Gender | a) Female b) Male |
| Marital status | 1. Single 2. Married / long-term partnership 3. Widowed 4. Separated / Divorced |
| Ethnicity | 1. Chinese 2. Malay 3. Indian 4. Mixed race: ____________________________ (please specify) 5. Others: ________________________________ (please specify) |
| Nationality | 1. Singaporean 2. Malaysian 3. Others: ________________________________ (please specify) |
| I identify with this faith-based group/ organization | 1. Buddhism or Taoism 2. Christianity or Roman Catholicism 3. Islam 4. Hinduism 5. Agnostic or Atheistic 6. Others: ________________________________ (please specify) |
| Education level | 1. Primary school 2. Secondary school 3. Diploma: _______________________________ (please specify) 4. Degree: ________________________________ (please specify) |
| Employment status | 1. Unemployed 2. Self-employed / Freelancer 3. Employee |
| My monthly income level | 1. $<5,000 2. $5,000 - <$10,000 3. $10,000 and above |
| Main Medical Diagnosis and prognosis  (List the top 2 life-limiting illnesses) | 1.  2. |
| **Part 2: In-Depth Interview with Patient +/- Nominated Healthcare Spokesperson** | |
| **Opening Question**   - Could you tell me more about the time a healthcare professional (e.g., doctor, nurse, social worker) spoke to you about your / your loved one’s care preferences for treatment and goals of care? [*probe: please tell me more about your experience*]   Or  “Can you recall any discussion conducted with the healthcare professionals regarding your/your loved one’s medical care/treatment plans/ goals of treatment in the past 7 days? [*probe: please tell me more about your experience*] | |
| ***the following questions in this segment need not be asked in the order given, but asked depending on how the participant answers the opening questions***  **People/Stakeholders:**   - Who spoke to you about this topic? - Do you remember who was present with you when the conversation took place? - [*Probe: “Who” can refer to the healthcare professional or family members, can probe if their presence at the conversation, made a difference in the conversation*]   **Setting:**   - Could you please tell me more about where this discussion took place? [*Probe: Can you think of where the discussion could have been conducted to make you feel more at ease?*]   **Content:**   - Could you tell me more about what was discussed? [*Probe: to go in detail about specifics like ICU/tube feeding/CPR/chemotherapy etc*] - If you have had this discussion more than once, how did the different conversations vary over time?   **Emotion**:   - How did the conversation make you feel then? Looking back, how do you feel about it now? [*Probe: Where did support come from?*] - Did you feel that it was an appropriate topic to discuss? Why or why not?   **Timing:**   - How do you feel about the time when your medical treatment preferences were discussed? [*Probe: If so did it matter the time it was conducted? “Time”: Can refer to exact time that GOC was discussed or timing in relation of the illness/disease trajectory*]   **Interviewing Skills**   - How would you rate the communication skill of the healthcare worker who had this conversation with you and Why? [*Probe: How was the discussion initiated with you? Was there anything that the healthcare worker did that made you feel more comfortable/ uneasy / could have done better? After this conversation, did your perception of the healthcare worker change in any way?*]   **Outcome**   - Was an agreement on your goals of care reached with the medical team? [*probe: If Yes, how?, if no why?*]   **Idea of a Good and Well Conducted Discussion**   - What would you tell your healthcare worker to guide him/her in leading these conversations in a better manner in the future? | |
| ***This may be a repetition of the opening question, but the aim is to give the participant some time to air any final thoughts after the going through the content of the interview***  **Summary Question**  In conclusion, please tell me how you would describe your experience of the GOC discussion? | |
